# Airway Epithelial Cells Drive Airway Smooth Muscle Cell Phenotype Switching to the Proliferative and Pro-inflammatory Phenotype

**DOI:** 10.3389/fphys.2021.687654

**Published:** 2021-07-06

**Authors:** M. J. O’Sullivan, J. H. Jang, A. Panariti, A. Bedrat, G. Ijpma, B. Lemos, J. A. Park, A. M. Lauzon, J. G. Martin

**Affiliations:** ^1^Meakins-Christie Laboratories, McGill University Health Centre, Montreal, QC, Canada; ^2^T.H. Chan School of Public Health, Harvard University, Boston, MA, United States

**Keywords:** epithelial cells, airway smooth muscle cells, proliferation, proinflammatory, miRNA-210

## Abstract

The increased mass of airway smooth muscle (ASM) in the airways of asthmatic patients may contribute to the pathology of this disease by increasing the capacity for airway narrowing. Evidence for the airway epithelium as a participant in ASM remodeling is accruing. To investigate mechanisms by which airway epithelial cells induce ASM cell (ASMC) proliferation, we have employed a co-culture model to explore markers of ASMC proliferative phenotype. Co-culture with epithelial cells led to incorporation of bromodeoxyuridine into ASMCs, indicating augmented proliferation and an associated increase in mRNA of the pro-proliferative co-transcription factor Elk1. Although the mitogen heparin-binding epidermal growth factor (HB-EGF) was augmented in the co-culture supernatant, the ASMC epidermal growth factor receptor (EGFR), an effector of HB-EGF induced proliferation, did not mediate epithelial-induced proliferation. The co-culture increased the expression of ASMC mRNA for the pro-inflammatory cytokines IL-6 and IL-8 as well as the pro-proliferative microRNA miR-210. The transcriptional repressor Max-binding protein (Mnt), a putative target of miR-210, was transcriptionally repressed in co-cultured ASMCs. Together, these data indicate that the airway epithelium-induced proliferative phenotype of ASMCs is not driven by EGFR signaling, but rather may be dependent on miR210 targeting of tumor suppressor Mnt.

## Introduction

Asthma is a chronic disease of the airways that is estimated to affect 300 million individuals globally (Eur. Resp. J, 1998). The pathophysiology of this disease involves airway wall remodeling including increased mass of smooth muscle (Dunnill et al., 1969; Ebina et al., 1993). Airway smooth muscle (ASM) plays a prominent role in airway constriction and is a key tissue in driving exacerbations. Increased ASM mass may be the most important factor contributing to increased airway resistance and excessive responsiveness to contractile agonists (Lambert et al., 1993). Several cellular sources of increased ASM have been postulated, including epithelial–mesenchymal transition (Berair et al., 2013), myofibroblast differentiation (Hautmann et al., 1997), and migration (Salter et al., 2017). An additional plausible source of increased ASM in the asthmatic airway is an increased proliferation of pre-existing ASM cells (AMSCs) (Ebina et al., 1993; Johnson et al., 2001; Woodruff et al., 2004).

Airway smooth muscle cells are phenotypically regulated such that they may exist in either a proliferative state or a contractile state. In culture conditions, serum deprivation induces the contractile phenotype (Halayko et al., 1999). The proliferative phenotype of ASMCs can be induced by transcription factor serum response factor (SRF) binding to Elk1 to transcribe *c-fos* (Wang et al., 2004). Furthermore, the interaction of SRF with Elk1 displaces SRF binding to myocardin, a smooth muscle specific co-transcription factor (Wang et al., 2004). Induction of the pro-proliferative phosphoinositide 3-kinase (PI3K)/protein kinase B (Akt) or mitogen-activated protein kinase (MAPK) pathways drives proliferation of ASMCs, and their induction represses the tension generated by smooth muscle tissue in response to activation by methacholine (Gosens et al., 2002). Akt signaling can induce the expression of the anti-apoptotic protein B-cell lymphoma 2 (Bcl-2) (Pugazhenthi et al., 2000).

Micro-RNA (miRNA) is a small (18–22 nucleotide) non-coding RNA that negatively regulates gene expression through binding to mRNA constructs preventing translation or targeting the mRNA for degradation. Several species of miRNA have been demonstrated to control smooth muscle phenotype. Myc is a proto-oncogene transcription factor that, along with its binding partner Max (Blackwood and Eisenman, 1991), drives the expression of genes associated with cell cycle progression. Myc drives proliferation in ASMCs (Volckaert et al., 2013) and may represent a therapeutic target for asthmatic myocytes. Max binding protein (Mnt) also binds to Max and antagonizes the activity of Myc:Max (Hurlin et al., 1997). Finally, in airway-derived fibroblast cultures, miR-210 has been shown to negatively regulate Mnt, leading to increased rates of proliferation (Bodempudi et al., 2014).

Another category of stimulus for proliferation of ASMCs that is of relevance to asthma pathology is pro-inflammatory cytokines. TNF-α induces ASMC methyl-[^3^H]thymidine incorporation in a PI3K-/Akt-dependent manner (Stamatiou et al., 2012), while CCL11 (Eotaxin), CCL5 (RANTES), IL-8, and MIP-1α all increased DNA synthesis in ASMCs (Halwani et al., 2011). The dual role of pro-inflammatory cytokines as effector molecules for recruiting leukocytes and directly driving airway remodeling is beginning to emerge. Recently, it has been demonstrated that airway epithelial cells in culture can induce proliferation of ASMCs (Malavia et al., 2009); however, the mechanism by which this occurs is largely unknown.

It is also increasingly evident that the airway epithelium plays an important role in driving airway remodeling in asthma (Lambrecht and Hammad, 2012). Airway epithelial cells can release ligands of the epidermal growth factor receptor (EGFR) as well as other mitogens, including heparin-binding epidermal growth factor-like growth factor (HB-EGF) (Hirota et al., 2012), amphiregulin, and transforming growth factor-β (TGF-β) (Kumar et al., 2004). The EGFR is upregulated in asthmatic airway epithelium (Puddicombe et al., 2000; Fedorov et al., 2005), is activated by Th2 cytokines (Heijink et al., 2007), and causes mucin secretion (Takeyama et al., 2001). EGFR signaling also plays a role in driving the proliferative response of smooth muscle in a rodent model of allergic asthma (Tamaoka et al., 2008). Although an increased rate of proliferation may be due to secreted growth factors from the epithelium, it is also possible that ASMC are phenotypically modulated and secrete mitogens that act in an autocrine manner. We wished to elucidate the molecular basis by which airway epithelial cells induce ASMC proliferation *in vitro* by exploring co-culture models. Due to the involvement of growth factor receptor signaling, and the novel role of miRNA in governing ASMC proliferation, we sought to examine the potential role of these molecules in epithelial-induced ASMC growth.

## Materials and Methods

### Reagents

Collagenase type IV from *Clostridium histolyticum* was obtained from Sigma-Aldrich (St. Louis, MI, United States). EGFR inhibitor tryphostin AG1478 (0.3 μM) was obtained from Cayman Chemical (Ann-Arbor, MI, United States). BrdU flow kit was obtained from BD Biosciences (Franklin Lakes, NJ, United States). EGFR inhibitor afatinib (0.5 μM) was obtained from Santa Cruz (Santa Cruz, CA, United States). qPCR primers for mRNA targets and Lipofectamine 2000 were obtained from Thermo Fisher Scientific (Waltham, MA, United States). qPCR primers for miRNA targets and miR-210 mimic and inhibitors were obtained from Exiqon (Vedbaek, Denmark).

### Cell Culture

Primary human ASMCs were obtained from lung transplant donors [International Institute for the Advancement of Medicine (IIAM)] or bronchial biopsies from patients with no history of lung disease. Micro-dissected tissue was digested overnight in collagenase (0.4 mg/ml) dissolved in Dulbecco’s modified Eagle’s medium (DMEM) containing streptomycin, penicillin, and amphotericin B (Anti-Anti; Thermo Fisher Scientific). The next day, the tissue was suspended in the digestion medium by gently passing through a serological pipette several times. Western blotting of markers of smooth muscle (α-smooth muscle actin and smooth muscle myosin) was used to confirm the cells were indeed ASMCs (data not shown). Cells were maintained in DMEM supplemented with Anti-Anti and 10% fetal bovine serum (FBS; Thermo Fisher Scientific). The medium was replaced every second day. For long-term storage, cells were cryo-stored in 90% FBS:10% dimethyl sulfoxide (DMSO) in liquid nitrogen. Prior to all experiments, the medium was changed to DMEM supplemented with 0.5% FBS containing Anti-Anti overnight prior to epithelial co-culture or stimulation with conditioned medium. Cells were studied between passages 2 and 6 and were used at approximately 80% confluence.

The bronchial epithelial cell line BEAS-2B cells were maintained similarly to ASMCs, serum deprived in DMEM containing 0.5% FBS with Anti-Anti, and studied when confluence was reached. Primary human bronchial epithelial cells (HBECs) were obtained from the CF Center Tissue Procurement and Cell Culture Core, University of North Carolina at Chapel Hill (courtesy of Dr. Randell) from donors with no history of lung disease. Cells were cultured for 14 days until well differentiated, as previously described (Lan et al., 2018). After 48 h in minimal medium (lacking hydrocortisone, EGF, and bovine pituitary extract), conditioned medium was collected and kept to be mixed 50:50 with 0.5% FBS in DMEM prior to incubation with ASMCs.

For co-culture experiments, Transwell^®^ permeable supports with 0.4-μm pores (Cat# 3460) (Corning, Corning, NY, United States) were utilized. Confluent epithelial cultures were serum deprived for 24 h prior to co-culture with ASMCs. For conditioned medium experiments, supernatant from serum-deprived confluent cultures was collected after 24 h of conditioning with fresh starvation medium, centrifuged at 1,500 RPM for 5 min and stored at −80°C.

### RT-qPCR

**FIGURE 1:**
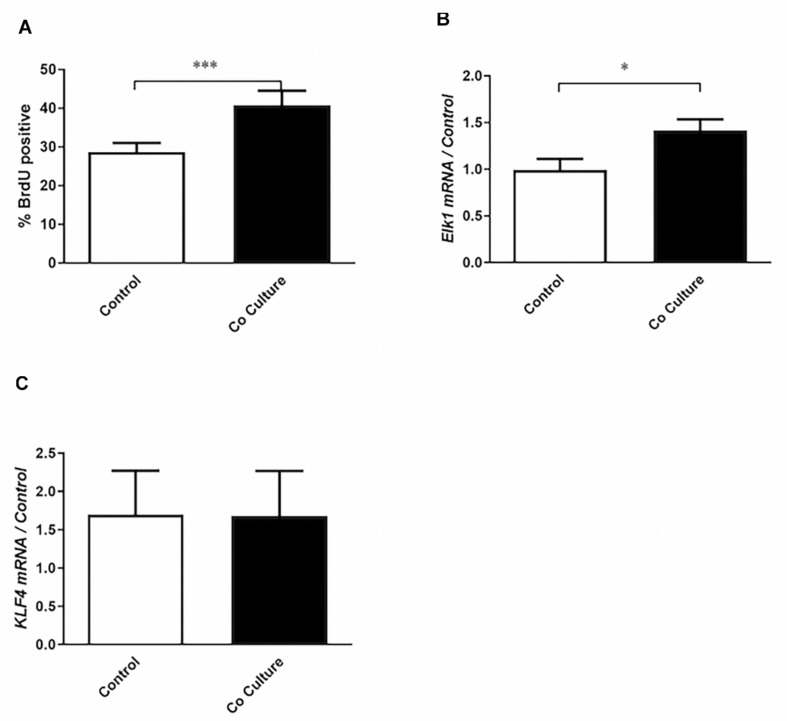
Co-culture with epithelial cells induces the proliferative phenotype in airway smooth muscle cells. **(A)** Airway smooth muscle cells (ASMCs) were cultured with (co-culture) or without (control) BEAS-2B cells for 24 h. Six hours after the initiation of co-culture, cells were pulsed with bromodeoxyuridine (BrdU). BrdU incorporation by flow cytometry was then performed to mark ASMCs that had entered into S-phase (*n* = 19). **(B,C)** RT-qPCR was performed on ASMCs to assess the expression of Elk1 (*n* = 7) and KLF4 (*n* = 6) after 24 h of co-culture with BEAS-2B cells, or without co-culture (control). Data are representative of means + 1 SE. Student’s paired *t*-test was utilized to compare groups. “*n*” refers to number of individual airway smooth muscle donors analyzed. ^∗^*P* < 0.05; ^∗∗∗^*P* < 0.001.

**FIGURE 2:**
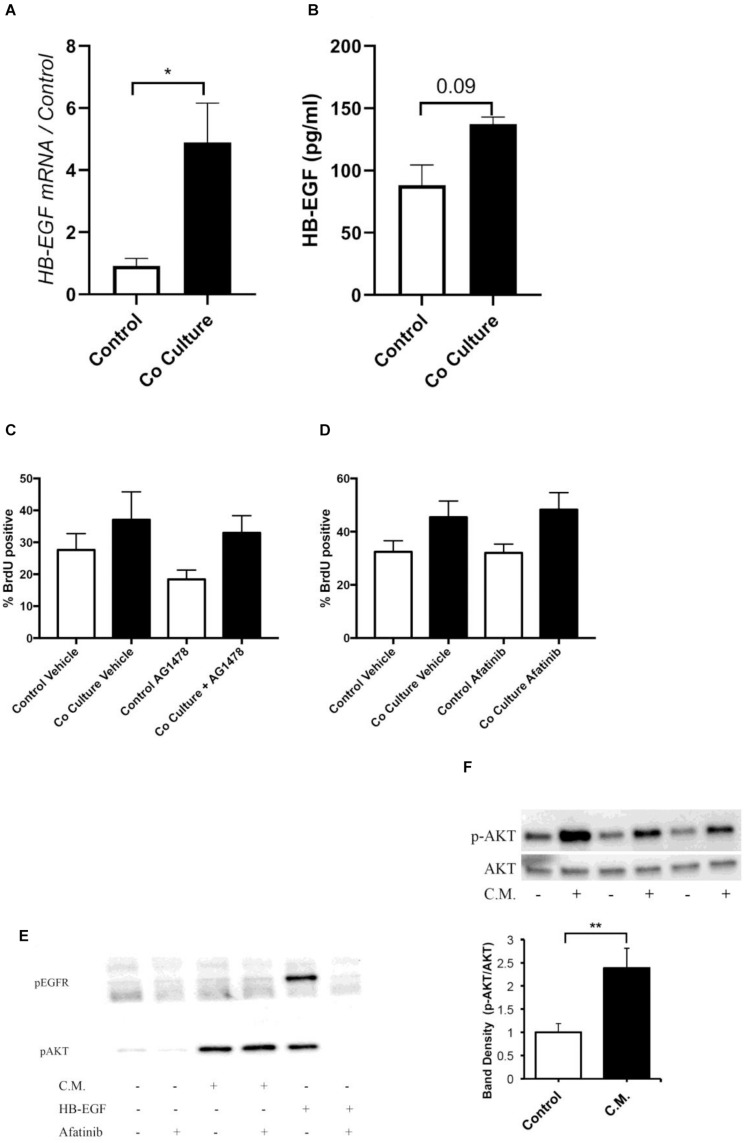
Co-culture induced proliferation is not mediated by an epidermal growth factor receptor (EGFR) ligand. **(A)** ASMC expression of heparin-binding growth factor receptor (HB-EGF) was examined by RT-qPCR 24 h after co-culture with or without (control) BEAS-2B cells (*n* = 4). **(B)** HB-EGF protein concentration was assayed in the cell culture supernatant of control or co-cultured ASMCs by ELISA after 24 h (*n* = 4). **(C,D)** Pre-treatment of ASMCs with 0.3 μM Tryphostin AG1478 (**C**, *n* = 4) or 0.5 μM afatinib (**D**, *n* = 5) did not prevent BEAS-2B induced proliferation of ASMCs. **(E)** Treatment for 30 min with conditioned medium of BEAS-2B cells did not induce phosphorylation of EGFR tyrosine 1173, however, AKT was phosphorylated by conditioned medium treatment. **(F)** Treatment for 5 min with conditioned medium of well-differentiated human bronchial epithelial (HBE) cells induced phosphorylation of AKT. Data are representative of means + 1 SE. ANOVA with Tukey’s *post hoc* pairwise comparisons were employed for comparisons with more than two groups, otherwise Student’s paired *t*-test was utilized. *P < 0.05; **P < 0.01.

**FIGURE 3:**
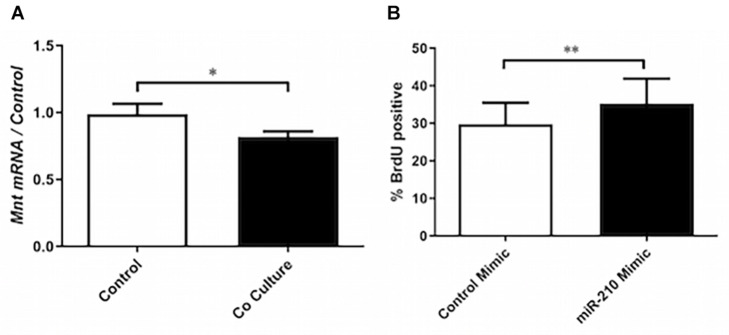
miR-210s role in co-culture induced proliferation. **(A)** RT-qPCR was performed on mRNA of ASM cells that had been co-cultured or not (control) with BEAS-2B cells for 24 h to assess the expression of Max-binding protein (Mnt) (*n* = 9). **(B)** ASM cells were transfected with a mimic of miR-210-3p or cel-miR-39-3p (control) prior to BrdU assay to assess the role of miR-210-3p in proliferation (*n* = 5). Data are representative of means + 1 SE. ANOVA with Tukey’s *post hoc* pairwise comparisons were employed for comparisons with more than two groups, otherwise Student’s paired *t*-test was utilized. ^∗^*P* < 0.05; ^∗∗^*P* < 0.01.

Airway smooth muscle cells were seeded at a density of 100,000 cells per well in six-well plates in growth medium. The next day, ASMCs were serum deprived for 24 h in starvation medium after which they were placed in co-culture with BEAS-2B cells. After 24 h of co-culture, ASMCs were washed once with PBS (Thermo Fisher Scientific), and mRNA was extracted using an RNeasy mini-kit (Qiagen, Valencia, CA, United States). Reverse transcription was performed on 100 ng of total RNA with AffinityScript qPCR cDNA synthesis kit (Agilent Technologies, Santa Clara, CA, United States). qPCR was performed using iTaq SYBR green supermix (Bio-Rad Laboratories, Hercules, CA, United States). Amplification of cDNA was performed using a StepOnePlus realtime PCR system (Applied Biosystems, Foster City, CA, United States) through 40 cycles with an annealing temperature of 60°C. Relative mRNA expression was calculated using the ΔΔCt method, and all gene expressions were normalized to S9 (Figures 1–3) or GAPDH.

For experiments examining miRNA expression, total RNA was extracted using Exiqon’s miRCURY RNA Isolation Kit–Cell and Plant according to the manufacturer’s protocol (Exiqon). miRNA cDNA libraries were generated with 20 ng of RNA using Exiqon’s Universal cDNA synthesis kit II, and RT-qPCR was performed with miRCURY LNA^TM^ Universal RT microRNA PCR kit according to the manufacturer’s protocol (Exiqon). Amplification of cDNA was performed using a StepOnePlus Real-Time PCR System (Applied Biosystems, Foster City, CA, United States). Relative mRNA expression was calculated using the ΔΔCt method, and all miRNA expressions were normalized to miR-103a-3p.

### mRNA Gene Array

Co-cultured or control ASMC-derived mRNA was analyzed for gene expression by HumanHT-12 version 4 Expression BeadChip Kit (Illumina, San Diego, CA, United States). Gene arrays were performed by Genome Quebec (Montreal, QC, Canada), and results were analyzed using FlexArray and cyber-T followed by Benjamini–Hochberg false discovery rate *P*-value correction for multiple comparisons. Raw data is available through the European Bioinformatics Institute, accession number E-MTAB-9004^1^. Gene ontology analysis was performed on enriched transcripts using PANTHER using Bonferroni correction for multiple testing.

### Micro-RNA Gene Array

Co-cultured or control ASMC-derived total RNA was analyzed for miRNA expression by miRCURY LNA^TM^ Array microRNA seventh generation profiling services (Exiqon). *P* value correction utilizing Benjamini–Hochberg false discovery rate was applied, and data analysis was conducted by Exiqon.

### RNA Sequencing

RNA was collected from ASMCs 24 h after stimulation with BEAS-2B conditioned media (CM). Illumina HiSeq4000 PE100 RNA sequencing was performed by Genome Quebec (Montreal, QC, Canada). The reads were aligned to the human reference genome GRCh37 using STAR (Spliced Transcripts Alignment to a Reference) (Dobin et al., 2013). Counts of genes were estimated using HtSeq-count (v0.10.0) (Anders et al., 2015) and normalized by implementing the TMM (trimmed mean of *M* value) method from the edgeR library (Robinson and Oshlack, 2010; Robinson et al., 2010). In this dataset, we only included genes at CPM above 1 in at least half of all the samples. The differentially expressed genes were selected by adjusting | Log2 FC| ≥ 0.5 and false discovery rate (FDR) corrected *P*-value (Benjamini–Hochberg) <0.05. Raw data are available through the European Bioinformatics Institute E-MTAB-9224^2^.

### Proliferation Assay

Airway smooth muscle cells were seeded in six-well plates at a density of 25,000 cells per well in growth medium. The following day, the medium was changed for starvation medium, 24 h later, ASMCs were either co-cultured with confluent BEAS-2B cultures that had been serum deprived for 24 h or not co-cultured. Six hours after the initiation of co-culture, BrdU was added to the culture medium according to the manufacturer’s protocol. Twenty-four hours after the initiation of co-culture, ASMCs were rinsed with PBS and collected using trypsin to fix and permeabilized for analysis of BrdU incorporation by anti-FITC-BrdU staining and flow cytometry according to the manufacturer’s protocol (BD Biosciences). As negative controls, ASMCs received growth medium to induce proliferation but were not incubated with BrdU. As positive controls, ASMCs received growth medium along with BrdU. Viable smooth muscle populations were selected for analysis, and gates were established based on negative and positive controls.

### MiR-210-3p Mimic and Inhibitor

Airway smooth muscle cells were seeded in six-well plates at a density of 25,000 cells per well in growth medium. The following day, cells were transfected with 50 nM of miR-210-3p inhibitor or mimic (Exiqon), along with 2 μl of Lipofectamine^®^ 2000 in 1 ml Opti-MEM (Thermo Fisher Scientific). As a control transfection for miR-210-3p mimic, cel-miR-39-3p (Exiqon) was transfected, and for miR-210-3p inhibitor controls, the anti-sense negative control A (Exiqon) was utilized. After 1 h, 1 ml of starvation medium without antibiotics was added to all wells. Six hours later, the medium was changed for fresh starvation medium containing antimicrobials. Transfection efficiency was assessed 48 h after addition of the inhibitors/mimics, and BrdU co-culture assays were performed from 48 to 72 h post-transfection.

### Heparin-Binding Epidermal Growth Factor Enzyme Linked Immunosorbent Assay

Cell culture supernatant was collected from ASMCs that had either been co-cultured with BEAS-2B cells or had not been co-cultured (control). Supernatant was centrifuged at 1,500 RPM for 5 min to remove any cells present, and the supernatant was assayed for HB-EGF with the Human HB-EGF DuoSet ELISA kit (R&D Systems, Minneapolis, MN, United States).

### Western Blot

After stimulation with epithelial-derived conditioned medium, AMSCs were washed with ice-cold PBS, and protein extraction was performed using protein lysis buffer containing 50 mM *Tris*HCl (pH 8), 150 mM NaCl, 1% NP-40, 0.5% sodium deoxycholate, and 0.1% SDS. The lysis buffer was supplemented with protease inhibitor cocktail (Sigma-Aldrich). Upon mechanical disruption, cell lysates were centrifuged at 13,000 RPM for 3 min, and proteins in the supernatant were measured by Quick Start Bradford Protein Assay (Bio-Rad). Proteins (20 μg) were loaded per lane into a separating gel. After separation, proteins were transferred to a PVDF membrane (Bio-Rad). Membranes were blocked with 5% bovine serum albumin (Sigma-Aldrich) for 1 h at room temperature prior to antibody incubation overnight at 4°C. A variety of primary antibodies were utilized: Phospho-Akt (Ser473) (D9E) (Cell Signaling Technology), total AKT (C67E7) (Cell Signaling Technology), and Phospho-EGF Receptor (Tyr1173) (53A5) (Cell Signaling Technology). Membranes were washed three times in *Tris*-buffered saline solution containing 0.1% TWEEN 20 (TBS-T). Membranes were incubated with secondary antibodies for 1 h at room temperature. Secondary antibodies included anti-mouse and anti-rabbit conjugated to horseradish peroxidase (Following three washes, antibodies were used at a concentration of 1–10,000).

### Statistical Analysis

Statistical analysis was carried out using GraphPad Prism 5 software (GraphPad Software Inc., San Diego, CA, United States). All data are presented as mean + 1 SE with ≥4 independent experiments. Experiments were considered independent when performed on ASMCs derived from different patients, or from different passage numbers measured on a different day. In experiments where >2 groups were compared, one-way ANOVA with Tukey’s *post hoc* test was utilized. For experiments where only two groups were compared, a paired Student’s *t*-test was employed. *P* values < 0.05 were considered to be significant.

## Results

### Co-culture With Epithelial Cells Induces the Proliferative Phenotype in Airway Smooth Muscle Cells

To determine if the bronchial epithelial cell line, BEAS-2B, increased the proliferation of ASMCs, the two cell types were co-cultured for 24 h prior to assessment of proliferation by BrdU incorporation. After co-culture with BEAS-2B cells, ASMCs demonstrated increased rates of proliferation (Figure 1A). Due to the role of the pro-proliferative co-transcription factor Elk1 in regulating ASMC phenotype, specifically in driving ASMC proliferation through activation of cyclin D1 (Chen and Khalil, 2006), we examined its mRNA expression and observed that it was increased after co-culture (Figure 1B). However, the mRNA expression of KLF4, another pro-proliferative co-transcription factor, was unchanged (Figure 1C). These results indicate that ASMCs are not only stimulated to proliferate by the epithelial co-culture but also differentially express a transcription factor associated with driving this phenotype.

### Co-culture-Induced Proliferation Is Not Mediated by an Epidermal Growth Factor Receptor Ligand

Due to the importance of mitogen stimulation in ASMC proliferation, we explored the EGFR as a potential target for mediating co-culture-induced proliferation. Within the ASMCs after co-culture, we examined the expression of the EGFR ligand HB-EGF, observing increases in this mRNA (Figure 2A). However, there was no significant increase in the concentration of this EGFR ligand at the protein level in the supernatant of co-cultured ASMCs (Figure 2B). Furthermore, pre-treatment with the tyrosine kinase inhibitors tryphostin AG1478 (3 μM) (Figure 2C) or afatinib (0.5 μM) (concentrations based on IC50 of respective inhibitors) (Figure 2D) did not prevent the induction of proliferation by co-culture as assessed by BrdU incorporation. Finally, stimulation of ASMCs with conditioned medium derived from BEAS-2B cells did not increase the phosphorylation of the EGFR on tyrosine 1173, although this medium did activate AKT (Figure 2E). Similarly, ASMCs stimulated with conditioned medium derived from well-differentiated HBECs activated AKT (Figure 2F). Other growth factor ligands produced by the epithelium, such as PDGF, will require exploration as potential mediators of this response.

### Co-culture Induces the Expression of Inflammatory Cytokines

**FIGURE 4:**
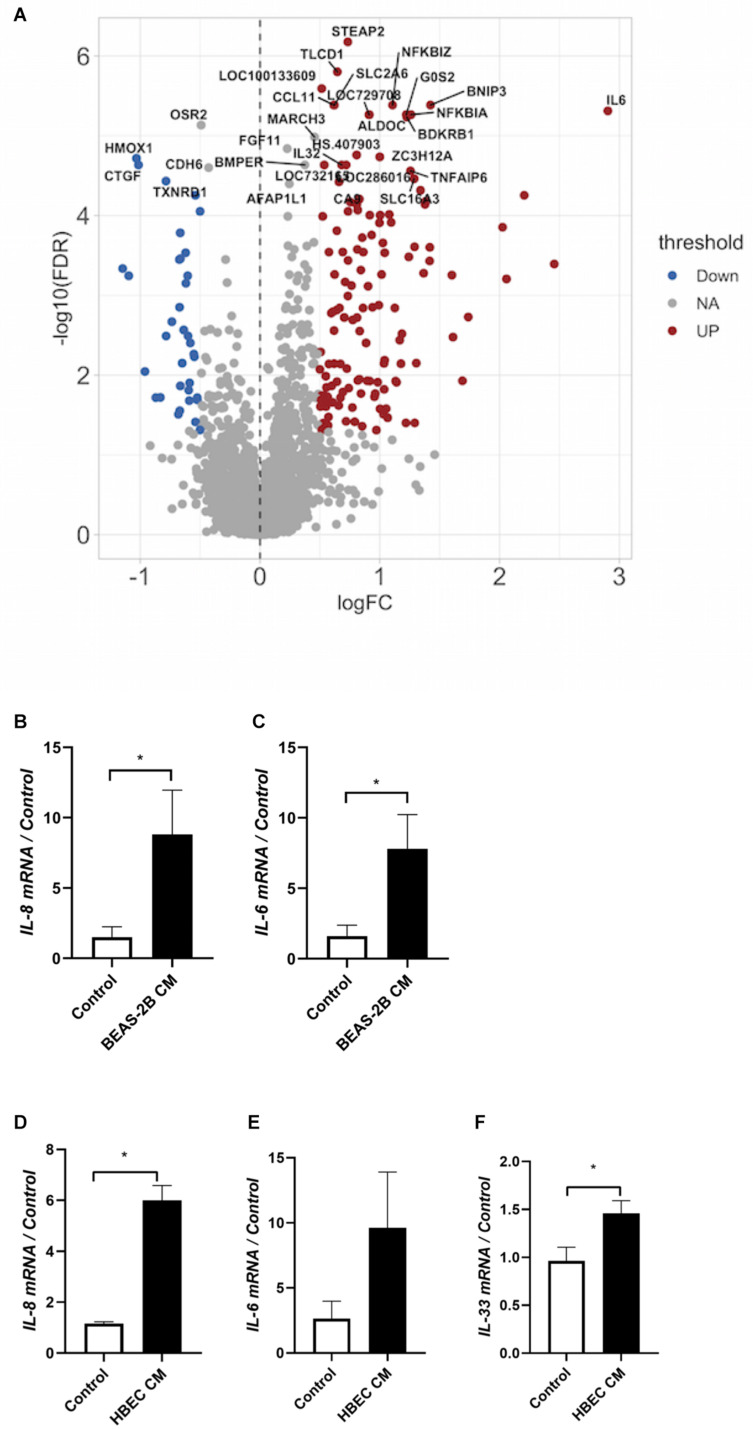
Co-culture induces the expression of inflammatory cytokines. **(A)** Messenger RNA was extracted from co-cultured or not co-cultured (control) ASMCs and gene array analysis was performed by Illumina HT-12 version 4 Expression BeadChip (Illumina) (*n* = 6). Gene array analysis was performed using FlexArray software and a Cyber-T test followed by a Benjamini–Hochberg *P*-value correction for multiple comparisons. The top 30 statistically significant genes are labeled on the volcano plot. Red indicates upregulation, blue indicates downregulation. FDR, false discovery rate; FC, fold change. **(B,C)** mRNA was extracted from ASMCs that had either been stimulated with conditioned medium from BEAS-2B cells, or stimulated with medium that was not conditioned by BEAS-2B cells. RT-qPCR was performed (*n* = 5). **(D–F)** mRNA was extracted from ASMCs that had either been stimulated with conditioned medium from well-differentiated human bronchial epithelial cells (HBECs) cultured at the air–liquid interface (ALI), or stimulated with medium that was not conditioned by HBECs. RT-qPCR was performed (*n* = 3). Data are representative of means + 1 SE. *P < 0.05.

To have a more complete understanding on how airway epithelial cells may augment ASMC proliferation, we ran a gene array exploring the differential expression of genes of ASMCs that had either been co-cultured with BEAS-2B cells or not. We observed significant increases in the expression of the pro-inflammatory cytokines CXCL1, IL-6, and IL-8 (the top 30 statistically significant genes are labeled on the volcano plot) (Figure 4A). Furthermore, there was a reduced expression of the anti-apoptotic protein Bcl2 after co-culture (Figure 4A). A total of 386 genes were differentially expressed, including 18 enriched genes associated with cellular metabolic processes and 10 genes associated with cellular response to stimulus. Furthermore, using the PANTHER enrichment test, biological processes including leukocyte chemotaxis and response to chemokine were significantly increased (*P* value 0.0384 and 0.0153, respectively). The list of genes with fold changes and *P*-values can be found online at https://www.ebi.ac.uk/arrayexpress/experiments/E-MTAB-9004/files/. BEAS-2B conditioned medium induced a pro-inflammatory phenotype in ASMCs as seen by the increases in IL-6 and IL-8 mRNA (Figures 4B,C), which was also induced by treatment of ASMCs with conditioned medium from well-differentiated, primary HBECs ASMCs (Figures 4D–F). These data further support the evidence indicating that ASMCs become more phenotypically skewed toward the proliferative/synthetic state after co-culture.

### Co-culture of Epithelial Cells Increases the Expression of miR-210-3p Without Affecting miR-143/145 or miR-25a

**FIGURE 5:**
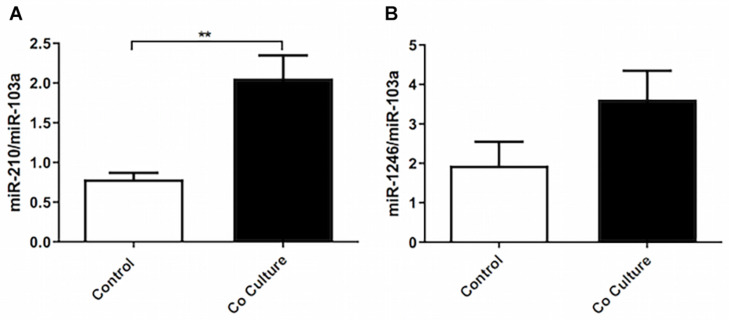
Co-culture of epithelial cells increases the expression of miR-210-3p without affecting miR-143/145 or miR-25a. Total RNA was extracted from co-cultured or not co-cultured (control) ASMCs and RT-qPCR was performed to examine the expression of **(A)** miR-210-3p (*n* = 9), and **(B)** miR-1246 (*n* = 9). Data are representative of means + 1 SE. For RT-qPCR, Student’s paired *t*-test was utilized. **P < 0.01.

To assess the expression of miRNAs previously identified to regulate ASMC phenotype, we co-cultured ASMCs with BEAS-2B cells and performed RT-qPCR. We observed no change in miR-143-3p/145-5p, nor in miR25 (data not shown). To further study differential miR expression in co-cultured ASMCs, we performed miRCURY LNA^TM^ Array microRNA seventh generation profiling (Exiqon). After Benjamini–Hochberg false discovery rate *P*-value correction for multiple comparisons, three candidate miRNAs were near significantly upregulated. To confirm the differential expression of these miRNAs, we performed RT-qPCR on co-cultured ASMCs, and observed a significant increase in miR-210-3p (Figure 5A), but did not confirm an increase in miR-1246 (Figure 5B) or miR-4732-5p (data not shown).

### MiR-210 Regulates Tumor Suppressor Mnt and Can Drive Proliferation *in vitro*

Due to miR-210’s previously reported role in driving fibroblast proliferation (Bodempudi et al., 2014), we explored the functional role of this miRNA in ASMCs. In co-cultured ASMCs, expression of miR-210’s putative target Max-binding protein (Mnt), a Myc inhibitor, was reduced (Figure 3A). Furthermore, transfection of ASMCs with miR-210 mimic increased the rate of proliferation of ASMCs, indicating a potential role for this miR in driving airway remodeling (Figure 3B). Although we did not explore the interaction of ELK-1 and Mnt, Myc, the target of Mnt, has been shown to be transcriptionally regulated by Elk1 (Hollander et al., 2016), suggesting another possible mechanism for increased proliferation.

### MiR-210 Inhibition Reduced IL-33 Expression in Airway Smooth Muscle Cells Stimulated With BEAS-2B Conditioned Medium

**FIGURE 6:**
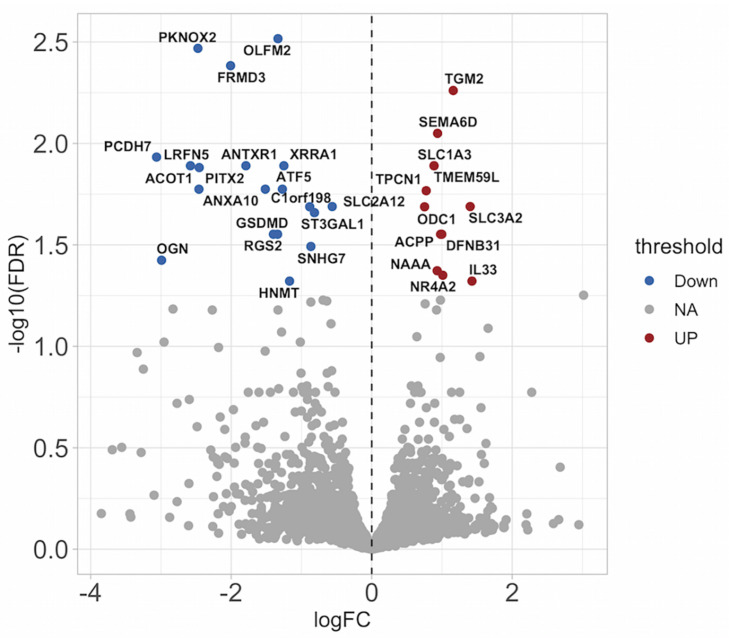
miR-210 inhibition causes differential gene expression in ASMCs stimulated with conditioned medium from BEAS-2B cells. RNA seq analysis was performed on ASMCs stimulated with conditioned medium (CM) from BEAS-2B cells in the absence or presence of a miR210-3p inhibitor. The top 31 differentially expressed genes are labeled. Red indicates upregulation, blue indicates downregulation. FDR, false discovery rate; FC, fold change.

To further explore the role of miR210, we stimulated ASMCs with BEAS-2B conditioned medium. ASMCs were transfected with either an miR210 inhibitor construct, or a non-specific control construct. mRNA was extracted 24 h after the incubation in conditioned medium, and RNA sequencing was performed. We wished to identify possible gene targets that were mediating miR210-induced proliferation. We found an upregulation of transglutaminase 2 (TGM2), a protein shown to be increased in induced sputum from asthmatic subjects (Hallstrand et al., 2010) and regulates eicosanoid production in airway epithelial cells (Hallstrand et al., 2012; Figure 6). Furthermore, there was less RGS2 in miR-210 inhibited ASMCs, which has been shown to be protective against the development of airway inflammation (George et al., 2018) and airway hyperresponsiveness (AHR) (Jiang et al., 2015). Also downregulated was Gasdermin D (GSDMD), a protein known to be involved in cell death through pyroptosis (Sborgi et al., 2016). The top 31 differentially expressed genes are labeled in the volcano plot. Future work examining the regulation of other proteins reported here is required for a more complete understanding of miR-210 biology. The entirety of the RNA seq results can be found online at https://www.ebi.ac.uk/arrayexpress/experiments/E-MTAB-9224/files/.

## Discussion

The purpose of this study was to examine mechanisms by which airway epithelial cells induce proliferation of ASMCs and affect the accompanying phenotype. We have demonstrated that ASMCs undergo increased rates of proliferation after co-culture with epithelial cells. In addition, the epithelium induces the expression of a number of pro-inflammatory cytokines. miR 210 is involved in mediating some of the phenotypic changes. Despite a strong rationale for exploring the EGFR in mediating ASM proliferation, our results do not support the role of HB-EGF or other EGFR ligands as mediators of the ASM proliferation.

In proximal airway biopsies obtained by bronchoscopy, ASM has been shown to be close to the epithelial layer (Pepe et al., 2005; Pretolani et al., 2017). It is therefore plausible that the proximity to the epithelium is such that pro-proliferative factors released by epithelial cells may have an impact on driving remodeling. Allergen-driven ASM remodeling in a rat model is critically dependent on the EGFR (Tamaoka et al., 2008; Siddiqui et al., 2013), and the airway epithelium has been shown to release several of its ligands in response to histamine (Hirota et al., 2012), leukotriene D4 (McGovern et al., 2010), and interleukin-13 (Allahverdian et al., 2008). Due to the potential of airway epithelial cells to secrete EGFR ligands (Kumar et al., 2004; Hirota et al., 2012), we explored the possibility that this tyrosine kinase receptor is responsible for mediating co-culture-induced proliferation. Although there was increased expression of mRNA for HB-EGF in ASMCs after co-culture as well as augmented protein in the supernatant, inhibition of the receptor with appropriate concentrations of AG1478 and afatinib did not affect the induced proliferation. The increase in expression of this EGFR ligand without an obvious functional role may imply that HB-EGF could act as a marker of the proliferative phenotype, but not necessarily drive proliferation itself. HB-EGF has been proposed previously as a biomarker of proliferating ASMCs, and the expression of this ligand in the muscle was associated with asthma severity (Hassan et al., 2010). A limitation of our study relates to the high isoelectric point of HB-EGF (Higashiyama et al., 1992) that can result in adsorption to surfaces such as plastic. This phenomenon may have reduced free concentrations sufficiently to prevent HB-EGF from exerting its effects.

A previous study has demonstrated that co-culture with epithelial cells drives the proliferation of ASMCs (Malavia et al., 2009). In this study, Malavia et al. explored the hypothesis that injury to epithelial cells may drive the proliferation of ASMCs. Asthmatic airway epithelial cells are likely more fragile than those derived from healthy controls (Trautmann et al., 2005). Injured epithelial cells induced a greater increase in ASMC proliferation than epithelial cells alone, an effect that may depend on matrix metalloproteinases (Malavia et al., 2009). The mechanism by which non-stimulated epithelial cells drive increased proliferation has remained elusive. Furthermore, the possibility of ASMCs participating in driving this proliferation in an autocrine manner has not been explored, and it has been demonstrated that this phenomenon is an important driver of proliferation (Johnson and Knox, 1999; Johnson et al., 2004).

Other recent work has demonstrated a role for micro-RNA (miR) in regulating ASMC proliferation (Hu et al., 2014). MiR-143 and miR-145 repress the expression of both Elk1 and another pro-proliferative co-transcription factor, Kruppel-like factor 4 (Klf4) (Cordes et al., 2009). Recently, miR-10a was demonstrated to inhibit ASMC proliferation by targeting the PI3K pathway, and this miR was shown to be the most abundantly expressed miR in ASMCs (Hu et al., 2014). Furthermore, miR-138 has been shown to regulate ASMC proliferation via targeted 3’-phosphoinositide-dependent kinase-1 (PDK1), a component of PI3K/Akt signaling (Liu et al., 2015). MiR-25, a miR that normally prevents KLF4 expression (Kuhn et al., 2010), was shown to be repressed in ASMCs by inflammatory cytokine stimulation. We observed increased miR-210-3p within ASMCs after co-culture with BEAS-2B cells. This is consistent with previous literature demonstrating that Elk1 signaling drives the expression of miRNA-210 (Kim et al., 2013; Liu et al., 2014). Previous work demonstrating a role of miR-210 in driving proliferation of fibroblasts in a Mnt-specific manner (Bodempudi et al., 2014) led us to explore this mechanism. Indeed, we observed reduced expression of Mnt in co-cultured ASMCs. Furthermore, other miRs have been associated with ASMC phenotype regulation. MiR143 and miR145 target the pro-proliferative co-transcription factor Elk1 (Cordes et al., 2009), and thus, we assessed whether these miRs were reduced after co-culture; however, we did not observe a diminished expression of these two miRs. Mir-25 targets KLF4 in ASMCs by regulating the proliferative phenotype (Kuhn et al., 2010). It was not anticipated that this miR would be differentially expressed by co-culture, as KLF4 mRNA was not increased in co-cultured cells. We suspect that these pro-inflammatory cytokines induced in ASMCs by HBECs act in concert with intracellular signaling molecules such as Mnt to induce proliferation of ASMCs. However, the direct impact of cytokine stimulation on Myc signaling in ASMCs is not well described. It has been shown that bromo- and extra-terminal (BET) family of proteins regulate both ASMC cytokine production and also associate with Myc to regulate cell proliferation (Perry et al., 2015). Further work inhibiting Mnt in ASMCs will lead to a better understanding of its role in proliferating ASMCs. We speculate that inhibition of Mnt with siRNA would lead to increased interactions between Max and Myc, thus, also driving the proliferative phenotype. Furthermore, other cytokines not explored here could participate in ASMC proliferation, for example, TNFα-induced ASMC proliferation; however, this response depends on IL-6 (Knobloch et al., 2016).

Smooth muscle cells differ from other muscles in their ability to exist along a spectrum of contractile and proliferative states (Owens, 1995). This ability to revert to a proliferative cell provides a potential explanation for the source of increased muscle mass surrounding the asthmatic airway. ASMCs harvested from asthmatic patients indeed have an increased proliferative capacity (Johnson et al., 2001). Although modeling studies indicate that the amount of asthmatic ASM is sufficient to explain increased airway responsiveness (Lambert et al., 1993; Macklem, 1996), it has been observed that ASMCs may exist in discrete populations, where there are cells actively proliferating at the same time as others that are expressing proteins of the contractile apparatus (Halayko et al., 1997). Furthermore, recent evidence shows that ASM force production is greater in asthmatic lungs when the ASM is sampled from the intrapulmonary region (Ijpma et al., 2020). The impact of the ASM architecture on airway narrowing will depend on its orientation and the extent to which it grows toward the airway lumen. A recent study has demonstrated that an increase in ASM mass does not disrupt the angle of the spiral the ASM makes around the airway (Ijpma et al., 2017). It has been noted that inwardly growing muscles would not only contribute to the increased mass observed in asthma but also may contribute to decreased luminal area (James et al., 1989; Janssen, 2012). It has been shown that ASM bundles remodel in the radial direction in the asthmatic airway and do not increase in bundle width, with no difference in the angle at which muscle bundles align along the airway axis (Ijpma et al., 2017).

Airway smooth muscle cell growth may represent an important mechanism by which the airway is remodeled in asthma, and understanding the mitogenesis of this tissue is an important area of research. Our aim was to better understand mechanisms by which structural cells of the airway interact with the anticipation that this approach might lead to future therapeutic targets for the treatment of airway diseases like asthma. New potential targets identified here include miR-210 and point away from the involvement of EGFR in this particular process. Confirmation of such results *in vivo* may prove informative and clarify the therapeutic relevance of such targets. Furthermore, studies examining co-culture of asthmatic HBECs with asthmatic ASMCs will provide a more comprehensive understanding of disease pathogenesis.

## Data Availability Statement

The datasets presented in this study can be found in online repositories. The names of the repository/repositories and accession number(s) can be found in the article/supplementary material.

## Author Contributions

MO’S, JJ, AP, and JM contributed to the design and conception of the study. MO’S, JJ, and AP conducted the experiments. MO’S, JJ, AP, and AB analyzed the data. MO’S and JM wrote the first draft of the manuscript. All authors contributed to manuscript revision, read, and approved the submitted version.

## Conflict of Interest

The authors declare that the research was conducted in the absence of any commercial or financial relationships that could be construed as a potential conflict of interest.

## Footnotes

1 https://www.ebi.ac.uk/arrayexpress/experiments/E-MTAB-9004

2 https://www.ebi.ac.uk/arrayexpress/experiments/E-MTAB-9224/

## References

[B1] Allahverdian S. Harada N. Singhera G. K. Knight D. A. Dorscheid D. R. (2008). Secretion of IL-13 by airway epithelial cells enhances epithelial repair via HB-EGF. *Am. J. Respir. Cell Mol. Biol.* 38 153–160. 10.1165/rcmb.2007-0173OC 17717322 PMC2214672

[B2] Anders S. Pyl P. T. Huber W. (2015). HTSeq–a Python framework to work with high-throughput sequencing data. *Bioinformatics* 31 166–169. 10.1093/bioinformatics/btu638 25260700 PMC4287950

[B3] Berair R. Saunders R. Brightling C. E. (2013). Origins of increased airway smooth muscle mass in asthma. *BMC Med.* 11:145. 10.1186/1741-7015-11-145 23742314 PMC3688527

[B4] Blackwood E. M. Eisenman R. N. (1991). Max: a helix-loop-helix zipper protein that forms a sequence-specific DNA-binding complex with Myc. *Science* 251 1211–1217. 10.1126/science.2006410 2006410

[B5] Bodempudi V. Hergert P. Smith K. Xia H. Herrera J. Peterson M. (2014). miR-210 promotes IPF fibroblast proliferation in response to hypoxia. *Am. J. Physiol. Lung. Cell Mol. Physiol.* 307 L283–L294. 10.1152/ajplung.00069.2014 24951777 PMC4137166

[B6] Chen G. Khalil N. (2006). TGF-beta1 increases proliferation of airway smooth muscle cells by phosphorylation of map kinases. *Respir. Res.* 7:2. 10.1186/1465-9921-7-2 16390551 PMC1360679

[B7] Cordes K. R. Sheehy N. T. White M. P. Berry E. C. Morton S. U. Muth A. N. (2009). miR-145 and miR-143 regulate smooth muscle cell fate and plasticity. *Nature* 460 705–710. 10.1038/nature08195 19578358 PMC2769203

[B8] Dobin A. Davis C. A. Schlesinger F. Drenkow J. Zaleski C. Jha S. (2013). STAR: ultrafast universal RNA-seq aligner. *Bioinformatics* 29 15–21. 10.1093/bioinformatics/bts635 23104886 PMC3530905

[B9] Dunnill M. S. Massarella G. R. Anderson J. A. (1969). A comparison of the quantitative anatomy of the bronchi in normal subjects, in status asthmaticus, in chronic bronchitis, and in emphysema. *Thorax* 24 176–179. 10.1136/thx.24.2.176 5821620 PMC471937

[B10] Ebina M. Takahashi T. Chiba T. Motomiya M. (1993). Cellular hypertrophy and hyperplasia of airway smooth muscles underlying bronchial asthma. A 3-D morphometric study. *Am. Rev. Respir. Dis.* 148 720–726. 10.1164/ajrccm/148.3.720 8368645

[B11] Fedorov I. A. Wilson S. J. Davies D. E. Holgate S. T. (2005). Epithelial stress and structural remodelling in childhood asthma. *Thorax* 60 389–394. 10.1136/thx.2004.030262 15860714 PMC1758889

[B12] George T. Chakraborty M. Giembycz M. A. Newton R. (2018). A bronchoprotective role for Rgs2 in a murine model of lipopolysaccharide-induced airways inflammation. *Allergy Asthma Clin. Immunol.* 14:40. 10.1186/s13223-018-0266-5 30305828 PMC6166284

[B13] Gosens R. Meurs H. Bromhaar M. M. McKay S. Nelemans S. A. Zaagsma J. (2002). Functional characterization of serum- and growth factor-induced phenotypic changes in intact bovine tracheal smooth muscle. *Br. J. Pharmacol.* 137 459–466. 10.1038/sj.bjp.0704889 12359627 PMC1573514

[B14] Halayko A. J. Camoretti-Mercado B. Forsythe S. M. Vieira J. E. Mitchell R. W. Wylam M. E. (1999). Divergent differentiation paths in airway smooth muscle culture: induction of functionally contractile myocytes. *Am. J. Physiol.* 276 L197–L206. 10.1152/ajplung.1999.276.1.L197 9887072

[B15] Halayko A. J. Rector E. Stephens N. L. (1997). Characterization of molecular determinants of smooth muscle cell heterogeneity. *Can. J. Physiol. Pharmacol.* 75 917–929. 10.1139/y97-106 9315361

[B16] Hallstrand T. S. Lai Y. Henderson W. R. Altemeier W. A. Gelb M. H. (2012). Epithelial regulation of eicosanoid production in asthma. *Pulm Pharmacol. Ther.* 25 432–437. 10.1016/j.pupt.2012.02.004 23323271 PMC3627426

[B17] Hallstrand T. S. Wurfel M. M. Lai Y. Ni Z. Gelb M. H. Altemeier W. A. (2010). Transglutaminase 2, a novel regulator of eicosanoid production in asthma revealed by genome-wide expression profiling of distinct asthma phenotypes. *PLoS One* 5:e8583. 10.1371/journal.pone.0008583 20052409 PMC2797392

[B18] Halwani R. Al-Abri J. Beland M. Al-Jahdali H. Halayko A. J. Lee T. H. (2011). CC and CXC chemokines induce airway smooth muscle proliferation and survival. *J. Immunol.* 186 4156–4163. 10.4049/jimmunol.1001210 21368236

[B19] Hassan M. Jo T. Risse P. A. Tolloczko B. Lemiere C. Olivenstein R. (2010). Airway smooth muscle remodeling is a dynamic process in severe long-standing asthma. *J. Allergy Clin. Immunol.* 125:e3. 10.1016/j.jaci.2010.02.031 20451038

[B20] Hautmann M. B. Madsen C. S. Owens G. K. (1997). A transforming growth factor beta (TGFbeta) control element drives TGFbeta-induced stimulation of smooth muscle alpha-actin gene expression in concert with two CArG elements. *J. Biol. Chem.* 272 10948–10956. 10.1074/jbc.272.16.10948 9099754

[B21] Heijink I. H. Marcel Kies P. van Oosterhout A. J. Postma D. S. Kauffman H. F. Vellenga E. (2007). Der p, IL-4, and TGF-beta cooperatively induce EGFR-dependent TARC expression in airway epithelium. *Am. J. Respir. Cell Mol. Biol.* 36 351–359. 10.1165/rcmb.2006-0160OC 17023689

[B22] Higashiyama S. Lau K. Besner G. E. Abraham J. A. Klagsbrun M. (1992). Structure of heparin-binding EGF-like growth factor. multiple forms, primary structure, and glycosylation of the mature protein. *J. Biol. Chem.* 267 6205–6212. 10.1016/S0021-9258(18)42682-8 1556128

[B23] Hirota N. Risse P. A. Novali M. McGovern T. Al-Alwan L. McCuaig S. (2012). Histamine may induce airway remodeling through release of epidermal growth factor receptor ligands from bronchial epithelial cells. *FASEB J.* 26 1704–1716. 10.1096/fj.11-197061 22247333

[B24] Hollander D. Donyo M. Atias N. Mekahel K. Melamed Z. Yannai S. (2016). A network-based analysis of colon cancer splicing changes reveals a tumorigenesis-favoring regulatory pathway emanating from ELK1. *Genome Res.* 26 541–553. 10.1101/gr.193169.115 26860615 PMC4817777

[B25] Hu R. Pan W. Fedulov A. V. Jester W. Jones M. R. Weiss S. T. (2014). MicroRNA-10a controls airway smooth muscle cell proliferation via direct targeting of the PI3 kinase pathway. *FASEB J.* 28 2347–2357. 10.1096/fj.13-247247 24522205 PMC3986841

[B26] Hurlin P. J. Queva C. Eisenman R. N. (1997). Mnt, a novel Max-interacting protein is coexpressed with Myc in proliferating cells and mediates repression at Myc binding sites. *Genes Dev.* 11 44–58. 10.1101/gad.11.1.44 9000049

[B27] Ijpma G. Kachmar L. Panariti A. Matusovsky O. Torgerson D. Benedetti A. (2020). Intrapulmonary airway smooth muscle is hyperreactive with a distinct proteome in asthma. *Eur. Respir. J.* 56 1902178. 10.1183/13993003.02178-2019 32299863

[B28] Ijpma G. Panariti A. Lauzon A. M. Martin J. G. (2017). Directional preference of airway smooth muscle mass increase in human asthmatic airways. *Am. J. Physiol. Lung. Cell Mol. Physiol.* 312 L845–L854. 10.1152/ajplung.00353.2016 28360113

[B29] James A. L. Pare P. D. Hogg J. C. (1989). The mechanics of airway narrowing in asthma. *Am. Rev. Respir. Dis.* 139 242–246. 10.1164/ajrccm/139.1.242 2912345

[B30] Janssen L. J. (2012). Airway smooth muscle as a target in asthma and the beneficial effects of bronchial thermoplasty. *J. Allergy* 2012:593784. 10.1155/2012/593784 23024662 PMC3457660

[B31] Jiang H. Xie Y. Abel P. W. Wolff D. W. Toews M. L. Panettieri R. A. (2015). Regulator of G-protein signaling 2 repression exacerbates airway hyper-responsiveness and remodeling in asthma. *Am. J. Respir. Cell Mol. Biol.* 53 42–49. 10.1165/rcmb.2014-0319OC 25368964 PMC4566111

[B32] Johnson P. R. Burgess J. K. Underwood P. A. Au W. Poniris M. H. Tamm M. (2004). Extracellular matrix proteins modulate asthmatic airway smooth muscle cell proliferation via an autocrine mechanism. *J. Allergy Clin. Immunol.* 113 690–696. 10.1016/j.jaci.2003.12.312 15100675

[B33] Johnson P. R. Roth M. Tamm M. Hughes M. Ge Q. King G. (2001). Airway smooth muscle cell proliferation is increased in asthma. *Am. J. Respir. Crit. Care Med.* 164 474–477. 10.1164/ajrccm.164.3.2010109 11500353

[B34] Johnson S. Knox A. (1999). Autocrine production of matrix metalloproteinase-2 is required for human airway smooth muscle proliferation. *Am. J. Physiol.* 277 L1109–L1117. 10.1152/ajplung.1999.277.6.L1109 10600880

[B35] Kim J. Park S. G. Song S. Y. Kim J. K. Sung J. H. (2013). Reactive oxygen species-responsive miR-210 regulates proliferation and migration of adipose-derived stem cells via PTPN2. *Cell Death Dis.* 4:e588. 10.1038/cddis.2013.117 23579275 PMC3641340

[B36] Knobloch J. Yanik S. D. Körber S. Stoelben E. Jungck D. Koch A. (2016). TNFα-induced airway smooth muscle cell proliferation depends on endothelin receptor signaling, GM-CSF and IL-6. *Biochem. Pharmacol.* 116 188–199. 10.1016/j.bcp.2016.07.008 27422754

[B37] Kuhn A. R. Schlauch K. Lao R. Halayko A. J. Gerthoffer W. T. Singer C. A. (2010). MicroRNA expression in human airway smooth muscle cells: role of miR-25 in regulation of airway smooth muscle phenotype. *Am. J. Respir. Cell Mol. Biol.* 42 506–513. 10.1165/rcmb.2009-0123OC 19541842 PMC2848741

[B38] Kumar R. K. Herbert C. Foster P. S. (2004). Expression of growth factors by airway epithelial cells in a model of chronic asthma: regulation and relationship to subepithelial fibrosis. *Clin. Exp. Allergy* 34 567–575. 10.1111/j.1365-2222.2004.1917.x 15080809

[B39] Lambert R. K. Wiggs B. R. Kuwano K. Hogg J. C. Pare P. D. (1993). Functional significance of increased airway smooth muscle in asthma and COPD. *J. Appl. Physiol.* 74 2771–2781. 10.1152/jappl.1993.74.6.2771 8365980

[B40] Lambrecht B. N. Hammad H. (2012). The airway epithelium in asthma. *Nat. Med.* 18 684–692. 10.1038/nm.2737 22561832

[B41] Lan B. Mitchel J. A. O’Sullivan M. J. Park C. Y. Kim J. H. Cole W. C. (2018). Airway epithelial compression promotes airway smooth muscle proliferation and contraction. *Am. J. Physiol. Lung. Cell Mol. Physiol.* 315 L645–L652. 10.1152/ajplung.00261.2018 30070589 PMC6295502

[B42] Liu S. Chuang S. M. Hsu C. J. Tsai C. H. Wang S. W. Tang C. H. (2014). CTGF increases vascular endothelial growth factor-dependent angiogenesis in human synovial fibroblasts by increasing miR-210 expression. *Cell Death Dis.* 5:e1485. 10.1038/cddis.2014.453 25341039 PMC4649533

[B43] Liu Y. Yang K. Sun X. Fang P. Shi H. Xu J. (2015). MiR-138 suppresses airway smooth muscle cell proliferation through the PI3K/AKT signaling pathway by targeting PDK1. *Exp. Lung Res.* 41 363–369. 10.3109/01902148.2015.1041581 26151666

[B44] Macklem P. T. (1996). A theoretical analysis of the effect of airway smooth muscle load on airway narrowing. *Am. J. Respir. Crit. Care Med.* 153 83–89. 10.1164/ajrccm.153.1.8542167 8542167

[B45] Malavia N. K. Raub C. B. Mahon S. B. Brenner M. Panettieri R. A. George S. C. (2009). Airway epithelium stimulates smooth muscle proliferation. *Am. J. Respir. Cell Mol. Biol.* 41 297–304. 10.1165/rcmb.2008-0358OC 19151317 PMC2742749

[B46] McGovern T. Risse P. A. Tsuchiya K. Hassan M. Frigola G. Martin J. G. (2010). LTD(4) induces HB-EGF-dependent CXCL8 release through EGFR activation in human bronchial epithelial cells. *Am. J. Physiol. Lung. Cell Mol. Physiol.* 299 L808–L815. 10.1152/ajplung.00438.2009 20889674

[B47] Eur. Resp. J. (1998). Worldwide variations in the prevalence of asthma symptoms: the international study of asthma and allergies in childhood (ISAAC). *Eur. Respir. J.* 12 315–335. 10.1183/09031936.98.12020315 9727780

[B48] Owens G. K. (1995). Regulation of differentiation of vascular smooth muscle cells. *Physiol. Rev.* 75 487–517. 10.1152/physrev.1995.75.3.487 7624392

[B49] Pepe C. Foley S. Shannon J. Lemiere C. Olivenstein R. Ernst P. (2005). Differences in airway remodeling between subjects with severe and moderate asthma. *J. Allergy Clin. Immunol.* 116 544–549. 10.1016/j.jaci.2005.06.011 16159622

[B50] Perry M. M. Durham A. L. Austin P. J. Adcock I. M. Chung K. F. (2015). BET bromodomains regulate transforming growth factor-beta-induced proliferation and cytokine release in asthmatic airway smooth muscle. *J. Biol. Chem.* 290 9111–9121. 10.1074/jbc.M114.612671 25697361 PMC4423696

[B51] Pretolani M. Bergqvist A. Thabut G. Dombret M. C. Knapp D. Hamidi F. (2017). Effectiveness of bronchial thermoplasty in patients with severe refractory asthma: clinical and histopathologic correlations. *J. Allergy Clin. Immunol.* 139 1176–1185. 10.1016/j.jaci.2016.08.009 27609656

[B52] Puddicombe S. M. Polosa R. Richter A. Krishna M. T. Howarth P. H. Holgate S. T. (2000). Involvement of the epidermal growth factor receptor in epithelial repair in asthma. *FASEB J.* 14 1362–1374. 10.1096/fasebj.14.10.1362 10877829

[B53] Pugazhenthi S. Nesterova A. Sable C. Heidenreich K. A. Boxer L. M. Heasley L. E. (2000). Akt/protein kinase B up-regulates Bcl-2 expression through cAMP-response element-binding protein. *J. Biol. Chem.* 275 10761–10766. 10.1074/jbc.275.15.10761 10753867

[B54] Robinson M. D. Oshlack A. (2010). A scaling normalization method for differential expression analysis of RNA-seq data. *Genome Biol.* 11:R25. 10.1186/gb-2010-11-3-r25 20196867 PMC2864565

[B55] Robinson M. D. McCarthy D. J. Smyth G. K. (2010). edgeR: a bioconductor package for differential expression analysis of digital gene expression data. *Bioinformatics* 26 139–140. 10.1093/bioinformatics/btp616 19910308 PMC2796818

[B56] Salter B. Pray C. Radford K. Martin J. G. Nair P. (2017). Regulation of human airway smooth muscle cell migration and relevance to asthma. *Respir. Res.* 18:156. 10.1186/s12931-017-0640-8 28814293 PMC5559796

[B57] Sborgi L. Ruhl S. Mulvihill E. Pipercevic J. Heilig R. Stahlberg H. (2016). GSDMD membrane pore formation constitutes the mechanism of pyroptotic cell death. *EMBO J.* 35 1766–1778. 10.15252/embj.201694696 27418190 PMC5010048

[B58] Siddiqui S. Novali M. Tsuchiya K. Hirota N. Geller B. J. McGovern T. K. (2013). The modulation of large airway smooth muscle phenotype and effects of epidermal growth factor receptor inhibition in the repeatedly allergen-challenged rat. *Am. J. Physiol. Lung. Cell Mol. Physiol.* 304 L853–L862. 10.1152/ajplung.00047.2012 23605002

[B59] Stamatiou R. Paraskeva E. Gourgoulianis K. Molyvdas P. A. Hatziefthimiou A. (2012). Cytokines and growth factors promote airway smooth muscle cell proliferation. *ISRN Inflamm* 2012:731472. 10.5402/2012/731472 24049651 PMC3767366

[B60] Takeyama K. Fahy J. V. Nadel J. A. (2001). Relationship of epidermal growth factor receptors to goblet cell production in human bronchi. *Am. J. Respir. Crit. Care Med.* 163 511–516. 10.1164/ajrccm.163.2.2001038 11179132

[B61] Tamaoka M. Hassan M. McGovern T. Ramos-Barbón D. Jo T. Yoshizawa Y. (2008). The epidermal growth factor receptor mediates allergic airway remodelling in the rat. *Eur. Respir. J.* 32 1213–1223. 10.1183/09031936.00166907 18653647

[B62] Trautmann A. Kruger K. Akdis M. Muller-Wening D. Akkaya A. Brocker E. B. (2005). Apoptosis and loss of adhesion of bronchial epithelial cells in asthma. *Int. Arch. Allergy Immunol.* 138 142–150. 10.1159/000088436 16179825

[B63] Volckaert T. Campbell A. De Langhe S. (2013). c-Myc regulates proliferation and Fgf10 expression in airway smooth muscle after airway epithelial injury in mouse. *PLoS One* 8:e71426. 10.1371/journal.pone.0071426 23967208 PMC3742735

[B64] Wang Z. Wang D. Z. Hockemeyer D. McAnally J. Nordheim A. Olson E. N. (2004). Myocardin and ternary complex factors compete for SRF to control smooth muscle gene expression. *Nature* 428 185–189. 10.1038/nature02382 15014501

[B65] Woodruff P. G. Dolganov G. M. Ferrando R. E. Donnelly S. Hays S. R. Solberg O. D. (2004). Hyperplasia of smooth muscle in mild to moderate asthma without changes in cell size or gene expression. *Am. J. Respir. Crit. Care Med.* 169 1001–1006. 10.1164/rccm.200311-1529OC 14726423

